# Quality Control in Routine Instrumental Epithermal Neutron Activation Analysis of Geological Samples

**DOI:** 10.6028/jres.093.024

**Published:** 1988-06-01

**Authors:** Maija Lipponen, Rolf Rosenberg

**Affiliations:** Technical Research Centre of Finland, Reactor Laboratory, SF-02150 Espoo, Finland

## Introduction

The Reactor Laboratory carries out analytical service by using neutron activation analysis. Altogether 50 elements are analyzed within a wide variety of geological, environmental, biological, archaeological, and industrial samples using instrumental and radiochemical thermal and epithermal neutron activation analysis. However, a major part of the analytical service is instrumental epithermal neutron activation analysis (IENAA) of geological samples and therefore only the quality control of this procedure will be discussed.

The main purpose for developing the technique was the need for a low cost and low detection limit determination of gold for geochemical exploration. The procedure used determines the concentration of 23 elements: Na, Sc, Cr, Fe, Co, Ni, Zn, As, Rb, Mo, Ag, Sn, So, Cs, Ba, La, Sm, Lu, Ta, W, Au, Th, U. A detection limit of 3 ppb for gold could only be obtained through IENAA. The required low cost and high capacity necessitated some compromises, and therefore an accuracy of ±10–20% was considered sufficient. The principal application field of the results (i.e., geochemical exploration) permits this.

Procedures for the simultaneous epithermal neutron irradiation of 600 samples and automatic measurement and data processing have been developed. A strong emphasis has been laid on the reliability of the results. This quality control has been twofold. The first stage was to show the procedure to be capable of producing results within the stated accuracy. The second stage is continuous control which ensures that the results of the routine analysis continue to fall within the stated error limits.

## The Analytical Procedures

### Standards

Four different standards are used to cover the 23 elements analyzed. Two of them are National rock standards produced by the Geological Survey of Finland. An aqueous solution of KBr is used as a standard for Br The fourth is a solid SiO_2_-based standard made at the Reactor Laboratory [1].

### Equipment

A Triga Mk II research reactor is used for the irradiations. The reactor is run about 7 h daily from Monday to Friday. The thermal flux is 1.2·10^12^ cm^−2^s^−1^ and the Cd-ratio for gold is 2. Twenty of the irradiation positions are used for thermal irradiations and 20 for epithermal irradiations. The epithermal flux is attained by using containers of aluminium 3 cm *ϕ*×25 cm in size, lined with 1 mm of cadmium and again with 0.2 mm of aluminium. These containers are permanently located in the reactor and are only taken up to change samples.

Measurements are performed with automatic γ- spectrometers comprising a Ge(Li) or a Ge detector with auxiliary electronics, a sample changer, a multichannel analyzer, a microcomputer, and input/output devices [2]. This system automatically measures a series of samples and simultaneously calculates the elemental concentrations that are printed on paper and cassette.

### Procedure for ENAA

The standards and powdered samples are weighed into polyethylene capsules with an inner volume of 0.5 mL. One irradiation series comprises four standards, 12 control samples, and 144 samples. These are inserted into cadmium containers and irradiated for 1 week.

The sample codes and weights are printed on floppy disks from which they are copied on a cassette for input into the computer memory at the start of a measurement series. After a decay time of 4–6 days, the samples are measured for 20 min/sample. Four series are measured in a week.

While this paper was being written, the computing and data input were centralized by connecting all the analyzers to one IBM/AT computer.

## Quality Control

### Development of Procedure

In developing the analytical procedure, the following aspects were considered: detection limits, cost, and accuracy. To decrease the cost, only a moderate accuracy, ±10–20%, was sought. The main consequences of this are: 1) there is no need to correct for the moderate flux variations between samples irradiated in one plane, and 2) a low activity and relatively short measurement time causing non-ideal counting statistics, is acceptable. In almost all other respects the maximum accuracy could be sought.

The possible errors include: sample representativity; contamination; weights; standards; flux variation; interfering nuclear reactions; neutron absorption; counting geometry; gamma-ray absorption; pulse pile-up, analyzer dead time; and interpretation of gamma-ray spectra.

The small sample size calls for a very homogeneous sample. This can be taken care of for all elements except gold. Additionally, contamination is a special problem to be addressed in the case of gold.

The standards are analyzed in our own laboratory using specially prepared liquid standards and earlier established standards. They are checked against international reference samples.

**Figure 1:**
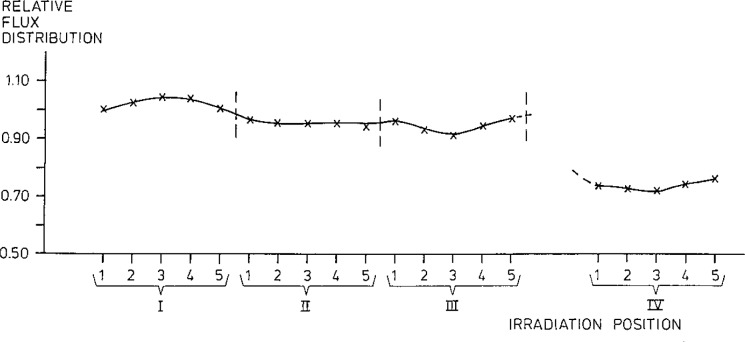
Flux variation in irradiation position.

Figure 1 shows the flux variation in the irradiation positions used. The average variation within the plane is ±3%. The horizontal variation within one container is about ±3%. The average flux difference between the layers of the samples is corrected for by using predetermined flux correction factors. Thus the average flux variation of individual samples relative to the standards is less than **±**10%.

With the exception of nickel, the analysis is based on (n;*γ*) reactions. The same radionuclides can be produced by the Fission of ^235^U, ^238^U and ^232^Th and by fast neutron reactions. Of the latter, only ^54^Fe(n;*α*)^51^Cr can constitute a serious source of error. The effect of Ti, ^46^Ti(n;p)^46^Sc, cannot be corrected without analyzing Ti separately, but it is negligible in most cases. The effect of the fission products is usually negligible, but has to be kept in mind.

With the small samples used, neutron absorption is negligible except in the case of gold. In average gold concentrations of less than 1 ppm, it has not been of significance, but in some cases intercomparisons with other analytical techniques have shown a 10% negative error in concentrations of 10 ppm or higher.

Errors in the counting geometry can be avoided by always Filling the capsules. The sample positioning, effected by means of the sample changer, is accurate.

Gamma-ray absorption is negligible with the small samples and relatively high gamma-energies used. With a few exceptions the sample activity is so low that the analyzer dead time is ~3%. Therefore the dead time correction is accurate and pulse pile-up negligible.

The statistical error is quite a good estimate of the total error in the peak intensity, as calculated by the computer program in use [3].

A set of USGS geochemical standards was analyzed to check the overall behaviour of the procedure [4]. The agreement was satisfactory.

## Quality Control of Routine Analysis

The purpose of this exercise is to assure that the analysis is continuously performed according to the criteria determined when the procedure was developed. Possible errors include: wrong standards; sample in wrong place during irradiation or measurement; Cd-lock of irradiation capsule ajar; wrong input data; malfunction during measurement; and too active a sample.

A number of control functions and samples are used to avoid the errors mentioned above or to find them after their occurrence.

**Figure 2:**
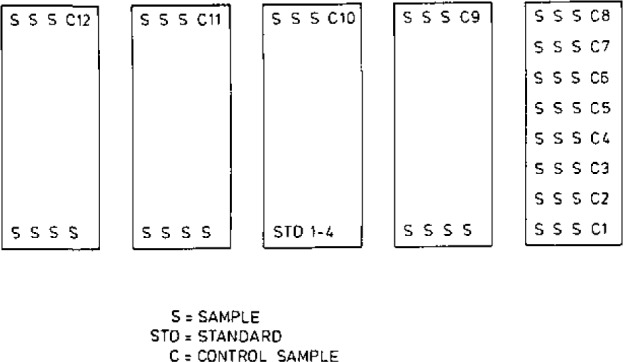
Irradiation configuration.

**Figure 3:**
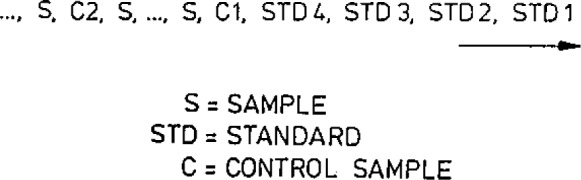
Measurement configuration.

Figure 2 shows the irradiation configuration and figure 3 the measurement configuration of one series of samples. The control samples are used to check that the samples are in the intended positions during irradiation so that the flux corrections will be right. It has happened that one sample tube is upside down during irradiation. Possible changes in the flux distribution due to other reasons can also be detected in this manner.

The results of the control samples are automatically compared with the “true values,” and if a difference greater than *±*10% is found, a warning is given. The results are then controlled manually to find out the reason for the error. A warning is also given if a sample is too active.

Table 1 gives typical long-term results of a control sample. The results of 33 samples were collected over a period of 6 months.

## Conclusions

IENAA has now been used for 7 years in the Reactor Laboratory. Annually 6000–1300 samples have been analyzed. The reliability of the results has been improved by increasing the number of control samples. The automated data control by the program has also been developed to prevent errors in advance and to aid in studying the results. Because most of the samples are analyzed for geochemical exploration, the acquired accuracy of ±10–20% is sufficient.

## Figures and Tables

**Table 1:** The mean value $\left(\bar{x}\pm S.D.\right)$ of 33 control samples and the calculated error from the recommended value. The sample is a rock standard produced by the Geological Survey of Finland

Element	Mean value (ppm)	Recommended value (ppm)	Relative Difference (%)
La	82±5	88	6.8
Sm	7.1±0.6	7.8	9.9
Fe	14400±1800	15600	7.7
Co	24.2±1.8	25	3.2
Na	21800±2000	22000	0.9
Sc	3.4±0.4	3.5	2.9
Ba	1090±100	1100	0.9
Cs	6.3±0.5	6.5	3.1
Rb	220±20	225	2.2
Ta	2.2±0.3	2.3	4.3
U	12.7±1.0	13	2.3
Th	52±3	53	1.9
